# Air–Liquid Interface (ALI) Exposure of Human Bronchial Epithelial Cells to Whole Gasoline Engine Exhaust Disrupts Autophagy and Proinflammatory Responses

**DOI:** 10.3390/toxics14030188

**Published:** 2026-02-24

**Authors:** Guoliang Li, Tao Yu, Xueyan Zhang, Wei Zhao, Min Zheng, Ying Qu, Bin Li, Ping Bin

**Affiliations:** 1 National Institute for Occupational Health and Poison Control, Chinese Center for Disease Control and Prevention, Beijing 100050, China; liguoliangahmu@163.com (G.L.); yutao@niohp.chinacdc.cn (T.Y.); zhangxy@niohp.chinacdc.cn (X.Z.); zhaowei@niohp.chinacdc.cn (W.Z.); zhengmin1@niohp.chinacdc.cn (M.Z.); quying@niohp.chinacdc.cn (Y.Q.); libin@niohp.chinacdc.cn (B.L.); 2 State Key Laboratory of Trauma and Chemical Poisoning, National Institute for Occupational Health and Poison Control, Chinese Center for Disease Control and Prevention, Beijing 100050, China

**Keywords:** autophagy, proinflammatory cytokines, NF-κB pathway, gasoline engine exhaust, human epithelial cells, air-liquid interface exposure

## Abstract

Gasoline engine exhaust (GEE) has been reported to contribute to the pathogenesis of pulmonary diseases. Autophagy, proinflammatory cytokines, and the NF-κB pathway core protein may play roles in the development of lung diseases caused by GEE. However, little is known about the possible toxic effects. Herein, we aimed to examine the crosstalk between GEE and the expression levels of autophagy-associated proteins (microtubule-associated proteins 1A/1B light chain 3A (LC3I/II)), proinflammatory cytokine genes (including *interleukin-1β* (*IL-1β*), *IL-6* and *IL-8*), and the NF-kB pathway core protein p65 by conducting an air–liquid interface exposure study in BEAS-2B cells. A CCK-8 assay was conducted to explore the viability of BEAS-2B cells exposed to GEE and 3-methyladenine (3-MA). The protein expression levels of LC3I/II and p65 were detected using Western blotting. The gene expression levels of *LC3B*, *IL-1β*, *IL-6*, and *IL-8* were measured using real-time PCR. We found that GEE decreased the viability of BEAS-2B cells in a dose-dependent manner, whereas 10%GEE exposure and 2.5 mM 3-MA had no significant effect. As the dose of GEE increased, LC3I/II protein and gene expression levels, proinflammatory cytokine gene expression levels, and p65 protein expression levels showed varying degrees of changes. Additionally, after treatment with 3-MA, these indicators tended to decrease, but only the gene expression levels of proinflammatory cytokines were statistically significant. These results suggest that GEE could interfere with autophagy and induce an inflammatory response in human bronchial epithelial cells, and that modest changes in autophagy could significantly alleviate this response, thereby providing new insights for the understanding of lung injury caused by GEE.

## 1. Introduction

Numerous epidemiological studies have found that exposure to traffic-related air pollution (TRAP) is associated with an increased risk of various respiratory diseases [1,2,3,4]. In particular, gasoline engine exhaust (GEE) is a major risk factor for TRAP in China. In our previous research, we found that GEE contains high concentrations of fine particulate matter, carbon monoxide, nitrogen oxides, and total hydrocarbons [5,6], which can reach the lungs directly and cause oxidative stress [7,8], inflammation in the lung epithelial cells [9,10], and even damage to genetic material [11]. Thus, exploring the potential molecular mechanisms by which GEE triggers lung injury via damage to epithelial cells is of great importance, as it could aid in the development of new effective therapeutic strategies.

Autophagy is involved in the development of various lung diseases, such as asthma, chronic obstructive pulmonary disease (COPD), acute pulmonary injury and respiratory infection [12]. Studies have shown that expression of the autophagy marker LC3 protein is increased in the sputum granulocytes, fibroblasts, neutrophils, peripheral blood eosinophils, and peripheral blood mononuclear cells of asthma patients [13,14,15,16]. Studies using human lung cells and tissues have reported that autophagy mediates the accumulation of challenge bodies composed of misfolded or damaged proteins, which, in turn, triggers chronic inflammatory and apoptotic responses, followed by the progression of emphysema [17,18]. Furthermore, mitochondrial autophagy may play a key role in the damage caused by environmental pollutants by removing damaged mitochondria and reducing excess ROS [19,20]; additionally, PM2.5 could induce the inflammatory response in rat spleen lymphocytes through autophagy activation of the NLRP3 inflammasome [21]. However, as a typical environmental pollutant, how GEE affects autophagy remains unknown.

Autophagy is an evolutionarily highly conserved process whereby biological macromolecules and organelles in cells are degraded and recovered. Autophagy plays a crucial role in maintaining lung homeostasis, including in regulating inflammatory responses [22]. Autophagy generally refers to macroautophagy, which involves the formation of double-membrane vesicles called “autophagosomes”, which can isolate components in cells such as damaged organelles, protein aggregates and misfolded proteins [23]. Cells form autophagosomes by encapsulating damaged organelles and proteins in double-membrane vesicles. These autophagosomes can bind to lysosomes to form autophagolysosomes, which then degrade and recover damaged organelles, misfolded proteins and invading pathogens, which are important inflammatory stimulators [24]. During acute lung infections caused by pathogens such as bacteria and viruses, autophagy may be activated as a protective mechanism. A previous study found that, in autophagy-deficient mice, the impaired clearance of pathogens led to an increased production of interleukin-1β (IL-1β), which, in turn, caused severe lung damage and reduced the survival rates [12]. During the development of COPD induced by cigarette smoke (CS), the expression of autophagy genes in the lung tissues of COPD patients increased, the accumulation of autophagy vesicles increased, and the autophagy marker microtubule-associated protein 1 light chain 3 (LC3) increased and was positively correlated with inflammatory cytokine levels [18,25]. Studies have demonstrated that PM exposure can promote cellular autophagy and inflammatory responses, while specific autophagy activators can aggravate PM2.5-induced inflammatory responses, such as the increase in the secretion of proinflammatory cytokines IL-6, IL-8, and tumor necrosis factor-α (TNF-α) [26]. Therefore, autophagy may play a dual role in the regulation of inflammatory responses. However, to date, no studies have examined the mechanism of autophagy in the inflammatory response caused by GEE.

Autophagy-related proteins (ATGs) participate in different steps of the autophagy process. The process of cell autophagy mainly includes four stages, namely, the formation of autophagy precursors, the formation of autophagosomes, the fusion of autophagosomes with lysosomes, and the degradation of autophagosome contents [23]. A bilayer membrane structure develops in the cytoplasm of cells in which autophagy is about to occur, called autophagy precursors, and then the autophagy precursor gradually forms cup-shaped depressions that encapsulate the cytoplasm, damaged/aging organelles, or misfolded proteins, finally forming autophagosomes [27]. This process of autophagosome formation is mainly guided by the microtubule-associated protein1 light chain 3 (MAP1LC3, also known as LC3). LC3 is sheared by ATG4 to form LC3I, and then it is combined with phosphatidyl ethanolamine to generate LC3-II and bind to the autophagosome membrane. LC3II is located on the autophagosome membrane and is widely used as a marker of autophagosome formation [27,28]. In summary, the LC3 protein is a marker of the formation of cell autophagosomes. Testing the expression of LC3I/II protein or *LC3B* gene can allow for an effective evaluation of the autophagy induced by GEE in bronchial epithelial cells.

Bronchial epithelial cells are the main cells of the human respiratory tract. They form a barrier that prevents pathogens and particles from entering the body. The general pathological response to inhaled harmful substances is the triggering of epithelial cell damage and acute inflammatory immune processes, leading to various respiratory diseases. Epithelial cells play a key role in regulating airway inflammation and can act as “effector” cells and participate in the inflammatory response in a variety of ways; additionally, abnormal epithelial cell function can affect local inflammatory responses and impair lung defense mechanisms [29]. The air–liquid interface (ALI) exposure system is an effective tool for studying the mechanism of GEE toxic effects in in vitro cell models [30]. The ALI exposure system can be used to simulate the mode of exposure of human bronchial epithelial cells to GEE, as it allows for exposure to all complete components of GEE without neglecting the gas phase. Therefore, the ALI exposure system is of great significance for in vitro research on the possible mechanisms of all components of GEE that induce autophagy and inflammatory responses.

IL-1β, IL-6 and IL-8 are all important proinflammatory cytokines. They tightly regulate cell-mediated immune responses and play a notable role in regulating cellular inflammatory responses. They are associated with the development of various non-infectious lung diseases, and their levels are positively correlated with the severity of these diseases [31,32]. The NF-κB pathway is considered a typical proinflammatory signaling pathway, and it can activate the transcription of various genes to regulate inflammation [33]. p65 is one of the transcriptional regulatory proteins of the NF-κB pathway; it functions as the activating component of the p65-p50 heterodimer and is able to bind to different transcriptional regulatory factors needed to activate NF-kB target genes [34]. Inhibiting NF-κB p65 phosphorylation can lead to potent anti-inflammatory effects in macrophages [35]. Overall, by analyzing the expression of these genes and/or protein levels, the inflammatory response status of cells can be better identified.

In vivo studies have reported that exposure to GEE and gasoline exhaust particles (GEPs) can induce significant inflammatory responses in the respiratory system of rats [36,37]; however, the specific mechanisms involved in the inflammatory response induced by GEE exposure require further study. 3-methyladenine (3-MA) is a classic, specific autophagy inhibitor. It can inhibit the formation of autophagosomes by inhibiting Class PI3K-III activity. Thus, it can inhibit cell autophagy, and reduce the expression of the autophagy protein LC3 [38]. It has been widely used as an autophagy inhibitor [39]. Therefore, this study treats bronchial epithelial cells with 3-MA before GEE exposure and then explores the effects of autophagy function, proinflammatory cytokines, and NF-κB signaling pathway-related proteins after GEE exposure. Thus, this study provides an important basis for further elucidating the role of autophagy in the GEE-induced inflammatory response and its toxicity mechanism.

In summary, this study employs ALI exposure technology to simulate the mode of exposure of bronchial airway epithelial cells (BEAS-2B) to GEE, and it examines the effect of GEE diluted at different ratios on the autophagy-related protein/gene expression levels (LC3I/II and *LC3B*), proinflammatory cytokine gene expression levels (*IL-1β*, *IL-6* and *IL-8*), and NF-κB pathway core protein expression levels (p65). To further study the role of autophagy in the GEE-induced inflammatory response, the cells are treated with 3-methyladenine before exposure to a 1:10 dilution of GEE, and the changes in the protein/gene expression levels of LC3I/II, *IL-1β*, *IL-6*, *IL-8*, and p65 are determined. These findings provide novel insights into and fundamental data on the role of autophagy in the inflammatory response induced by GEE in bronchial cells, and they may contribute to the development of strategies for the treatment of GEE-related respiratory disorders.

## 2. Materials and Methods

### 2.1. Cell Culture

BEAS-2B cells are immortalized cells derived from normal human bronchial epithelium. They were purchased from BeNa Culture Center (BNCC, Beijing, China) and authenticated via STR profiling (see our previous work [11]). The BEAS-2B cells were cultured in Dulbecco’s Modified Eagle Medium (DMEM) (Gibco, Grand Island, NY, USA), supplemented with 10% fetal bovine, penicillin and streptomycin (100 U/mL) (Gibco), and maintained in a constant-temperature incubator (Thermo scientific, Waltham, MA, USA) at 37 °C, 5% CO_2_, and maximal relative humidity.

### 2.2. Gasoline Engine Exhaust (GEE) Preparation and Cell Treatment

#### 2.2.1. Clean Air Collection

After connecting a 0.20 µm filter (PN4251, Pall Corporation, Port Washington, NY, USA), clean air consisting of 21% O_2_ and 79% N_2_ (Beijing Oriental Medical Gas Co., Ltd., Beijing, China) was collected in a 20 L Tedlar bag (Beijing Safelab Technology Ltd., Beijing, China). This was carried out before the experiment.

#### 2.2.2. Collection and Dilution of Gasoline Engine Exhaust (GEE)

GEE samples were obtained using a two-wheeler motorcycle. First, the motorcycle was subjected to cold-start conditions and idled for 5 min, and then GEE samples were collected using a Tedlar bag. The exhaust was diluted with clean air to obtain different concentrations, including 5%GEE (1__GEE_:20__clean air_), 10%GEE (1__GEE_:10__clean air_), 20%GEE (1__GEE_:5__clean air_), and 100%GEE. The particulate matter, nitrogen oxide, and total hydrocarbon concentrations in these GEE samples were as reported in our previous work [6,40]. The clean air was used as a control group. All samples were collected before the experiments.

#### 2.2.3. Cell Treatment with Gasoline Engine Exhaust (GEE) and 3-Methyladenine (3-MA)

Cell treatment with GEE was performed using the air–liquid interface exposure system established by Li [41], where BEAS-2B cells were directly exposed to GEE. Briefly, cells ((4–5) × 10^5^ per insert) in 1 mL suspension were seeded on porous membranes. After culturing for 24 h, the inserts with the cells were transferred to the ALI exposure device (HRH-CES1332, Beijing Huironghe Science and Technology Co., Beijing, China) and exposed to clean air or different concentrations of GEE at a flow rate of 10 mL/min/well for 1 h.

Furthermore, to observe the effects of an autophagy inhibitor on autophagy protein and inflammatory markers induced by GEE, cells were treated with 3-methyladenine (3-MA) before exposure to clean air or GEE. In order to select the appropriate dose, exposure to 5 concentrations, namely, 0.5 mM, 1.25 mM, 2.5 mM, 5 mM, and 10 mM, was carried out for 4 h to pre-evaluate the cytotoxicity of the BEAS-2B cells; then, we chose a dose and incubated the cells for 24 h to observe differences in between the clean air and GEE exposure groups.

### 2.3. Cell Viability Assay

The cell viability of BEAS-2B was measured using a Cell Counting Kit-8 (CCK-8) (Tong Ren Chemical Research Institute, Kumamoto, Japan), which was performed as previously described by our laboratory [11]. In brief, after exposure to clean air or GEE exposure was over, 1 mL of CCK-8 working solution (1_-CCK-8 solution_:9_-serum-free medium_) was added to the porous membranes in each insert and then incubated at 37 °C and 5% CO_2_ for another 1 h. The supernatants were transferred to a 96-well plate for absorbance (A) measurements using a microplate reader (Infinite M200 PRO, TECAN, Hombrechtikon, Switzerland) at 450 nm. Relative cell survival (%) = (A _exposed group_ − A _cell-free group_)/(A _blank control group_ − A _cell-free group_) × 100%.

### 2.4. Analysis of Gene Expression of LC3B and Inflammatory Cytokines

The gene expression levels of *LC3B* and inflammatory cytokines (*IL-1β*, *IL-6,* and *IL-8*) were determined using a real-time PCR analysis. Details of the primer sequences can be found in Table 1. After the purity and quantity of the extracted RNA were assessed using an ND-2000 spectrophotometer (Thermo Fisher Scientific, Waltham, MA, USA), the total RNA was converted into complementary DNA (cDNA) using a reverse transcription kit (Shanghai Yisheng Biotechnology Co., Shanghai, China), and the cDNA was subsequently amplified and quantified using the iQ5 Real-Time PCR Detection System (Bio-Rad Laboratories, Inc., Hercules, CA, USA). The relative gene expression levels were analyzed using the 2^−ΔΔCt^ method. The normalized expression level of *IL-1β*, *IL-6*, *IL-8*, and *LC3B* mRNA was determined relative to that of *Gapdh*.

### 2.5. Western Blot Analysis

In the initial dose–effect observation, the protein expression levels of LC3I/II and p65 were measured at two designated time points: immediately after 1 h of exposure to GEE and allowing an additional 24-h incubation after 1 h of exposure to GEE. After the exposure dosage was selected, these measurements were only carried out immediately after 1 h of exposure to GEE. Following exposure to clean air or GEE, RIPA lysis buffer was used to completely disrupt the cells, and they were quantified using the bicinchoninic acid assay (BCA) method according to the instructions (Beyotime Biotechnology Inc., Shanghai, China). The total lysate protein was electrophoretically separated on a 10% SDS-PAGE gel. After membrane transfer, the membrane was sealed with 5% skim milk powder at room temperature for 1 h. The target bands were incubated with primary antibodies specific to LC3I/II (1:1000, Abcam, Cambridge, UK), p65 (1:1000, Abcam) and GAPDH (1:10,000, Abcam) overnight at 4 °C. After conjugation with secondary antibodies at room temperature for 1 h and washing the membrane for 30 min, a fully automatic chemiluminescence image analysis system (Tanon 5200, Shanghai Tianneng Technology Co., Shanghai, China) was used to visualize the protein bands. The quantified data were normalized to glyceraldehyde-3-phosphate dehydrogenase (GAPDH). The relative expression of each target protein was measured using the ratio of the grayscale values of the target protein and GAPDH protein bands, and then a semi-quantitative analysis was performed.

### 2.6. Statistical Analysis

Data are expressed as means ± SD, and groups were compared using a one-way ANOVA. The LSD method was used to compare the variances between groups, and the Dunnetts-T3 method was used to compare the variances evenly. All tests were two-sided, and the significance level was set to less than 0.05.

## 3. Results

### 3.1. Gasoline Engine Exhaust Decreases Viability of BEAS-2B Cells

**Figure 1:**
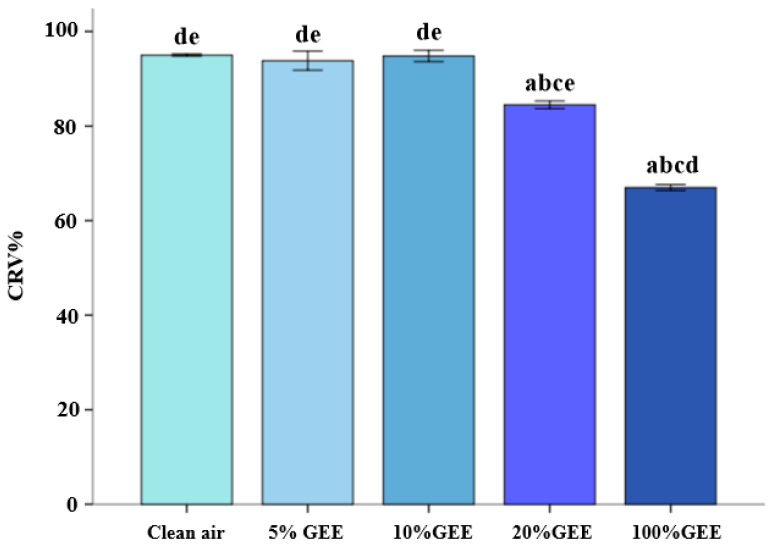
Relative viability of the BEAS-2B cells in different concentrations of GEE. Note: n = 6. a: Compared with the clean air group, *p* < 0.05. b: Compared with the 5%GEE group, *p* < 0.05. c: Compared with the 10%GEE group, *p* < 0.05. d: Compared with the 20%GEE group, *p* < 0.05. e: Compared with the 100%GEE group, *p* < 0.05. Abbreviations: GEE, gasoline engine exhaust; CRV: cell relative viability.

As shown in Figure 1, the relative survival rates of the BEAS-2B cells decreased in a dose-dependent manner after 1 h exposure to GEE. A significant decrease was observed in the 20%GEE and 100%GEE groups (both *p* < 0.05). Considering the significant damage to the BEAS-2B cells, 10%GEE was selected as the appropriate exposure concentration for the next experiment.

### 3.2. Effect of Gasoline Engine Exhaust on Autophagy in BEAS-2B Cells

**Figure 2:**
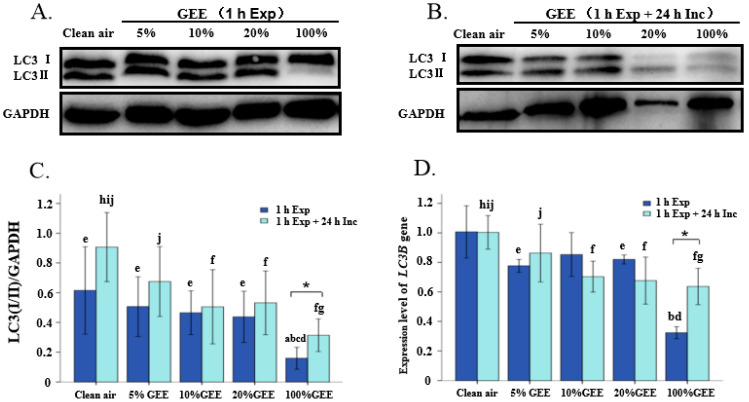
Protein and gene expression levels of LC3 in the BEAS-2B exposed to different concentrations of GEE. (**A**) Representative Western blot images of LC3 protein. (**B**) Representative Western blot images of LC3 protein. (**C**) Relative expression levels of LC3 protein. (**D**) Relative expression levels of *LC3B* gene. Note: a, f: Compared with the clean air group, *p* < 0.05. b, g: Compared with the 5%GEE group, *p* < 0.05. c, h: Compared with the 10%GEE group, *p* < 0.05. d, i: Compared with the 20%GEE group, *p* < 0.05. e, j: Compared with the 100%GEE group, *p* < 0.05. *: Compared between 1 h exposure to GEE and 1 h exposure to GEE + 24 h incubation, *p* < 0.05. Abbreviations: GEE, gasoline engine exhaust; Exp, exposure; Inc, incubation.

A Western blot analysis showed that, after 1 h exposure, only the 100%GEE group had a significant difference from the clean air group in terms of the protein expression levels of LC3I/II, and it showed significantly lower levels than the other GEE exposure groups, namely, the 5%GEE, 10%GEE, and 20%GEE groups (all *p* < 0.05). After 1 h exposure + 24 h incubation, the protein expression level of LC3I/II in the 10%GEE, 20%GEE, and 100%GEE groups was significantly lower than that in the clean air group (all *p* < 0.05), and it was significantly lower in the 100%GEE group than in the 5%GEE group. A comparison between the results obtained after 1 h exposure and 1 h exposure + 24 h incubation revealed that there was a significant difference in the expression level of LC3I/II (*t* = −2.435, *p* = 0.031), but the level was still lower than that in the clean air group. These results are shown in Figure 2C.

After 1 h exposure, the gene expression level of *LC3B* in the 100%GEE group was significantly lower than that in the clean air, 5%GEE, and 20%GEE groups (all *p* < 0.05). After 1 h exposure + 24 h incubation, the gene expression level of *LC3B* showed a gradually decreasing trend, and it notably decreased in the 10%GEE, 20%GEE, and 100%GEE groups compared with in the clean air group (all *p* < 0.05). Additionally, when comparing the 1 h exposure and 1 h exposure + 24 h incubation groups, it was found that the group without 24 h incubation had a lower level of *LC3B* gene expression (*t* = −4.822, *p* = 0.009). All of these results are shown in Figure 2D.

Based on the changes in LC3I/II protein and gene expression levels in BEAS-2B cells, it was determined that exposure to GEE can affect autophagy by decreasing the protein and gene expression levels of LC3I/II, especially exposure to high concentrations of GEE. Following 1 h exposure + 24 h incubation, the protein and gene expression levels of LC3I/II increased, and these results suggest that the cells might have initiated repair functions to maintain their homeostasis after autophagy-related proteins and genes were damaged.

### 3.3. Gene Expression of Proinflammatory Cytokines Induced by Gasoline Engine Exhaust in BEAS-2B Cells

**Figure 3:**
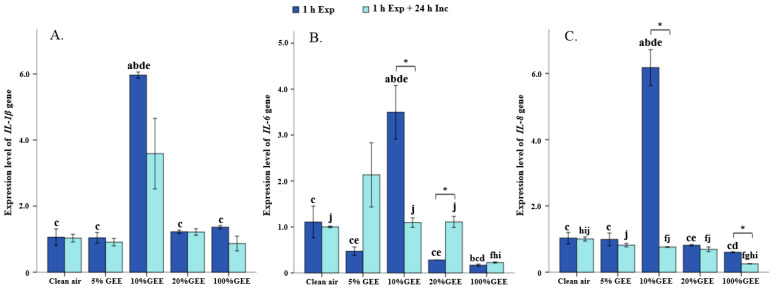
Gene expression levels of proinflammatory cytokines in the BEAS-2B exposed to different concentrations of GEE. (**A**) Relative expression levels of *IL-1β*. (**B**) Relative expression levels of *IL-6*. (**C**) Relative expression levels of *IL-8*. Note: a, f: Compared with the clean air group, *p* < 0.05. b, g: Compared with the 5%GEE group, *p* < 0.05. c, h: Compared with the 10%GEE group, *p* < 0.05. d, i: Compared with the 20%GEE group, *p* < 0.05. e, j: Compared with the 100%GEE group, *p* < 0.05. *: Compared between 1 h exposure to GEE and 1 h exposure to GEE + 24 h incubation, *p* < 0.05. Abbreviations: GEE, gasoline engine exhaust; Exp, exposure; Inc, incubation.

This study also explored the expression levels of various genes, including *IL-1β*, *IL-6*, and *IL-8* (Figure 3). The results showed that the gene expression levels of the proinflammatory cytokines IL-1β, IL-6, and IL-8 were the highest after 1 h exposure to 10% GEE, but they tended to decrease after the cells were incubated for another 24 h (all *p* < 0.05). In addition, the expression levels of these three proinflammatory cytokines were significantly decreased in the 100%GEE exposure group (all *p* < 0.05). Taken together, these results suggest that exposure to low concentrations of GEE can stimulate inflammatory responses in BEAS-2B cells, while exposure to high concentrations of GEE can inhibit such responses.

### 3.4. Protein Expression of p65 Induced by Gasoline Engine Exhaust in BEAS-2B Cells

**Figure 4:**
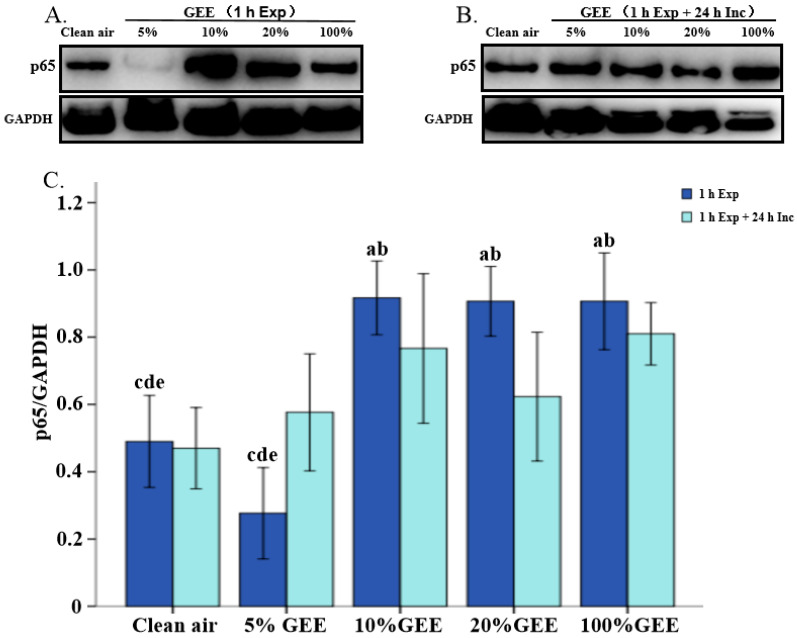
Protein expression levels of p65 in the BEAS-2B exposed to different concentrations of GEE. (**A**) Representative Western blot images of p65 protein. (**B**) Representative Western blot images of p65 protein. (**C**) Relative expression levels of p65 protein. Note: a: Compared with the clean air group, *p* < 0.05. b: Compared with the 5%GEE group, *p* < 0.05. c: Compared with the 10%GEE group, *p* < 0.05. d: Compared with the 20%GEE group, *p* < 0.05. e: Compared with the 100%GEE group, *p* < 0.05. Abbreviations: GEE, gasoline engine exhaust; Exp, exposure; Inc, incubation.

In order to confirm the impact of GEE on the NF-κB signaling pathway, we examined the levels of its core protein, p65. We found that GEE promoted p65 expression (Figure 4). The protein expression levels of p65 in the 10%GEE, 20%GEE, and 100%GEE groups was significantly higher than that in the clean air group (all *p* < 0.05). When the cells were incubated for another 24 h, no statistical differences were observed between the groups (all *p* > 0.05). These results indicate that GEE exposure can also affect the NF-κB signaling pathway.

### 3.5. Effect of 3-Methyladenine (3-MA) on LC3I/II, Proinflammatory Cytokine and p65 Expression in BEAS-2B Cells Exposed to GEE

#### 3.5.1. Effect of 3-MA Pretreatment on the Relative Viability of BEAS-2B Cells

**Figure 5:**
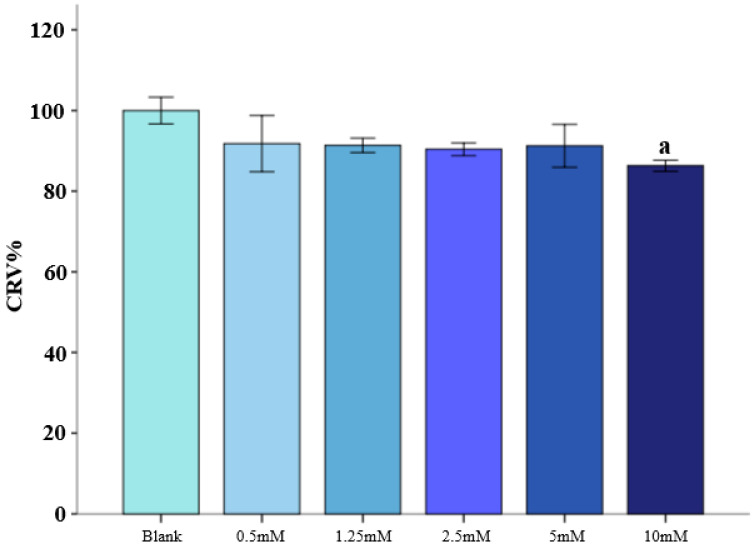
Relative cell viability with varying concentrations of 3-MA treatment. Note: a: Compared with the blank control group, *p* < 0.05.

As shown in Figure 5, the survival rate of the BEAS-2B cells tended to decrease as the concentration of 3-MA increased. Except for the 10mM group, the relative survival rates of the cells in the other groups were above 90%. Compared with the blank control group, the relative survival rate of the BEAS-2B cells in the 10mM group significantly decreased (*p* < 0.05).

**Figure 6:**
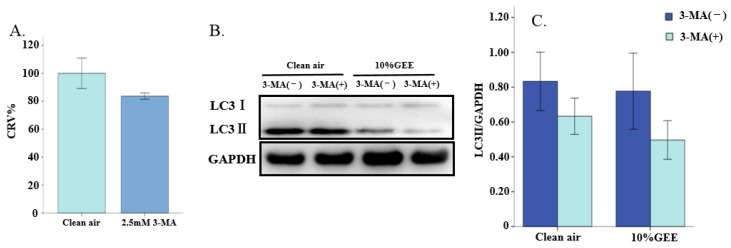
Effects on the LC3I/II protein through 3-MA in the BEAS-2B cells. (**A**) Cell relative viability with clean air or 2.5mM 3-MA treatment. (**B**) Representative Western blot images of LC3 with 3-MA treatment. (**C**) Relative expression levels of LC3 protein. Abbreviations: GEE, gasoline engine exhaust; 3-MA, 3-methyladenine.

As shown in Figure 6A, when using 2.5 mM 3-MA to incubate the BEAS-2B cells for 24 h, no significant differences were found in cell viability compared with the clean air group. The relative cell viability was higher than 80%. This suggested that this dose did not have significant adverse effects on relative cell viability and that it could be used for subsequent experiments in BEAS-2B cells.

#### 3.5.2. Effect of 3-MA on the Protein Expression of LC3I/II in BEAS-2B Cells Exposed to GEE

According to the Western blot analysis results (Figure 6B), although 3-MA did not have a significant inhibitory effect on the LC3I/II protein in the BEAS-2B cells (all *p* > 0.05), the expression of this autophagy-related protein decreased in both the clean air group and the GEE exposure groups (Figure 6C). These results suggested that 2.5 mM 3-MA may exacerbate the GEE-induced inhibition of autophagy.

#### 3.5.3. Effect of 3-Methyladenine (3-MA) on Inflammatory Cytokine Gene Expression in BEAS-2B Cells Exposed to GEE

**Figure 7:**
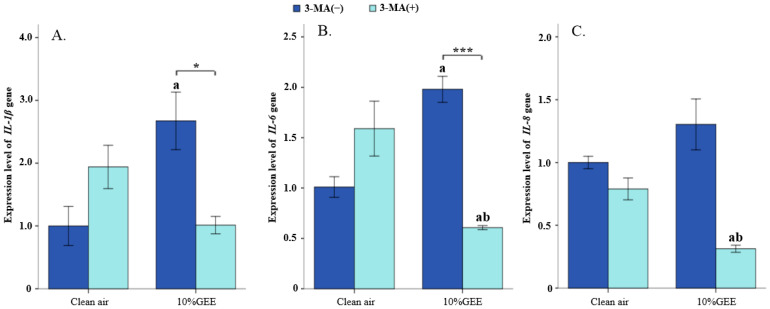
Effects on inflammatory cytokine gene expression levels through 3-MA in the BEAS-2B cells. (**A**) Relative expression levels of *IL-1β*. (**B**) Relative expression levels of *IL-6*. (**C**) Relative expression levels of *IL-8*. Note: a: Compared with the clean air + 3-MA(−) group, *p* < 0.05. b: Compared with the clean air + 3-MA(+) group, *p*< 0.05. *: Compared between 3-MA(−) and 3-MA(+) group, *p* < 0.05. ***: Compared between 3-MA(−) and 3-MA(+) group, *p* < 0.001. Abbreviations: GEE, gasoline engine exhaust; 3-MA, 3-methyladenine.

Figure 7 shows the changes in inflammatory cytokines (including *IL-1β*, *IL-6*, and *IL-8*) in the BEAS-2B cells exposed to clean air and 10%GEE for 1 h after pretreatment with 3-MA. The gene expression level of *IL-1β* in the 10%GEE exposure group significantly increased compared to that in the clean air without 3-MA group (*t* = 3.024, *p* = 0.039); however, after pretreatment with 3-MA, it significantly decreased (*t* = −3.466, *p* = 0.026). The gene expression levels of *IL-6* and *IL-8* in the 3-MA treatment groups were significantly lower than those in both clean air groups (all *p* < 0.05), and, compared with the 10%GEE group without 3-MA treatment, the gene expression level of *IL-6* decreased significantly in the 10%GEE + 3-MA group (*t* = 10.519, *p* < 0.001). These results indicate that 2.5 mM 3-MA contributes to decreasing the proinflammatory response caused by GEE in the BEAS-2B cells following ALI exposure.

#### 3.5.4. Effect of 3-Methyladenine (3-MA) on the Core Protein Expression of NF-κB Pathway in BEAS-2B Cells Exposed to GEE

**Figure 8:**
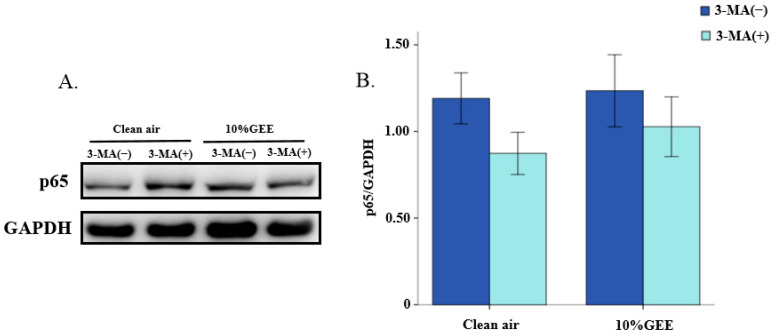
Effect of 3-methyladenine on the p65 protein expression in the BEAS-2B cells. (**A**) Representative Western blot images of p65 protein. (**B**) Relative expression levels of p65 protein. Abbreviations: GEE, gasoline engine exhaust; 3-MA, 3-methyladenine.

Figure 8 shows the expression levels of p65, the core protein of the NF-κB pathway, with or without 2.5 mM 3-MA pretreatment for 24 h. GEE did not have a significant effect on the core proteins of the NF-κB pathway following 2.5 mM 3-MA treatment (*p* > 0.05). However, in both the clean air group and 10%GEE exposure groups, the protein expression level of p65 protein tended to decrease. These results suggest that 3-MA has an alleviating effect on the GEE-induced expression of the core protein in the NF-κB pathway.

## 4. Discussion

Gasoline cars have always been a popular means of transportation in China, especially in the northern region. Therefore, gasoline engine exhaust (GEE) represents a major risk factor for traffic-related air pollution (TRAP). GEE contains thousands of toxic chemicals that can reach the microairways and alveoli, exerting various effects, including inducing oxidative stress and inflammatory responses in animal models [8]. In humans, exposure to automobile exhaust and fuel vapour can impair lung function in a time-dependent manner [42]. In this study, using an air–liquid interface exposure approach, we investigated whether in vitro exposure to GEE was associated with alterations in the levels of autophagy, proinflammatory cytokines, and the NF-κB pathway core protein p65 in bronchial epithelial cells (BEAS-2B). Further, autophagy inhibitors (3-MA) were used to treat cells before GEE exposure, and then the impact of GEE on the cellular inflammatory response was observed. In addition, we also compared the expression levels of autophagy, inflammatory cytokines, and p65 at two designated time points, namely, immediately after the 1 h of GEE exposure and allowing an additional 24-h incubation after 1 h of exposure to GEE. All results are particularly interesting.

As the primary respiratory organ and a major target of ambient airborne pollutants, the pulmonary system is commonly used to assess GEE toxicity in vitro. To date, almost no one has examined the impact of GEE on autophagy in the body. Therefore, we investigated the potential toxicity of GEE via autophagy function in bronchial epithelial cells. The results indicate that GEE lead to a clear dose–response decrease in autophagy, which manifested as a decrease in LC3I/II protein and *LC3B* gene expression levels, and that exposure to high concentrations of GEE can significantly inhibit autophagy. This result is similar to that found in a study on exposure to traffic-related diesel pollution particles [43]. Simultaneously, the overall trends in autophagic responses were consistent as the exposure concentration increased at both of the two designated time points. In the high-dose group (100%GEE), there was a statistically significant difference in the expression levels of autophagy protein genes at these two time points, with them rebounding after 24 h incubation. These results indicate that autophagy is a highly dynamic process that might be finely regulated by multiple signaling pathways. However, the underlying regulatory mechanisms remain to be further explored.

In our previous study, it was found that GEE contributed to increasing levels of cellular proinflammatory cytokines, including IL-1β and IL-6, in in vitro models, such as A549 cell lines [44]. In the present work, this result was confirmed at the level of gene expression. Increased production of inflammatory cytokines (i.e., *IL-1β* and *IL-6*) was induced by 10%GEE; additionally, p65 protein expression increased when the BEAS-2B cells were exposed to 10%GEE, 20%GEE, and 100%GEE. These results suggest that GEE exposure and uncontrolled autophagy can lead to enhanced inflammation.

As a key mechanism in cell degradation and recycling, autophagy plays a complex role in the regulation of inflammation. In recent years, studies have found that inhibiting autophagy may reduce the levels of inflammatory cytokines through specific pathways, especially in chronic inflammatory diseases [45]. Inhibiting autophagy can reduce the exocytosis of NLRP3 and its stability, thereby inhibiting inflammasome activation and the secretion of cytokines such as IL-1β. This mechanism was verified in a macrophage model, indicating that autophagy inhibition may reduce inflammation by regulating the NLRP3 degradation pathway [46]. In our current study, pretreatment of cells with 3-MA, an autophagy antagonist, indicated only a weak inhibitory effect on autophagy; this was determined based on the LC3I/II protein levels in the BEAS-2B cells after exposure to GEE, in which there was no statistically significant difference. However, in this state, autophagy was not completely suppressed, and it was necessary to determine the relationship between the secretion levels of proinflammatory cytokines and autophagy in the cell model; this would allow us to better ascertain the role of autophagy in the inflammatory response after exposure to GEE. To explore the possible mechanism involved in regulating the secretion of proinflammatory cytokines, we pretreated the BEAS-2B cells with 2.5 mM 3-MA and found that the inflammatory cytokine gene and p65 protein expression levels in the 10%GEE exposure group were reduced. In particular, *IL-1β* and *IL-6* were significantly decreased, which suggests that moderate suppression of autophagy can reduce the risk of proinflammatory reactions. Similar studies have found that inflammation can be effectively controlled by inhibiting autophagy [47,48]. These data suggest an intimate relationship between autophagy and anti-inflammatory mechanisms via reductions in inflammatory cytokines such as interleukins. Therefore, this study further examined the relationship between autophagy and inflammatory responses following GEE exposure, and it obtained more information on the mechanism of inflammatory effects caused by GEE. However, determining the exact mechanisms of how GEE impacts humans requires further research.

IL-1β, IL-6, and IL-8 are three important cytokines that play key roles in inflammatory and immune responses, and there are complex interactions between them [49]. IL-1β is a core proinflammatory cytokine and a key upstream driver. It often acts as an “initiator” or “amplifier” in inflammatory responses. IL-1β can directly induce the production of IL-8 and indirectly promote the release of IL-6. IL-6 acts as a pleiotropic amplifier and cooperates with IL-1β to amplify the inflammatory response, while IL-8 mainly acts as an effector mediator, and its expression is directly regulated by IL-1β. This network of interactions is critical in diseases such as inflammation, infection, and cancer [50]. Therefore, detection of these cytokines can help evaluate disease activity and treatment response. More importantly, our present results suggest that inflammatory cytokines were at their lowest levels after exposure to high concentrations of GEE or following profound autophagy inhibition after exposure to low concentrations GEE. These results indicate that cells with dysfunctional autophagy will appear to be immune-tolerant and will be unable to recognize the invasive effects of xenobiotic chemicals, which, in turn, will affect the recruitment of inflammatory cytokines and may lead to the deterioration of defense mechanisms. However, the underlying mechanisms remain to be further explored.

## 5. Conclusions

In conclusion, our research findings reveal that air–liquid interface (ALI) exposure to all components of gasoline engine exhaust (GEE) disrupted autophagy and inflammatory responses in BEAS-2B cells. Additionally, 2.5 mM 3-methyladenine (3-MA) could effectively reduce the expression of proinflammatory cytokines induced by 10%GEE at the genetic level, especially that of *IL-1β* and *IL-6*. This study enriches the theory of lung injury induced by GEE, providing a potential new insight for the treatment of GEE-induced damage. Nonetheless, the mechanisms of GEE-induced lung injury still require further investigation.

## 6. Limitations

This study also has some limitations. First, we only evaluated the results obtained after 1 h of exposure to GEE and initially identified the role of GEE in regulating autophagy and inflammatory responses. We still need to conduct chronic toxicity tests with long-term repeated exposure to GEE, which would better reflect daily human exposure. Second, we only used immortalized cells to explore the impact of GEE on autophagy and inflammatory effects at the protein and gene levels; we did not examine primary cells or conduct in vivo validation. Finally, only a limited number of autophagy and inflammatory markers were examined, and flux assays are still required. The relationship between autophagy and inflammatory responses is complex, and it may play an important role in the carcinogenic toxicity of GEE; thus, further studies in these areas are needed, especially regarding potential mechanisms.

## Figures and Tables

**Table 1:** PCR primer sequence for gene expression.

Gene	Forward Primer (5′-3′)	Reverse Primer (5′-3′)
*Gapdh*	GGTCACCAGGGCTGCTTT	GGACTCCACGACGTACTCA
*LC3B*	TTCAGGTTCACAAAACCCGC	TCTCACACAGCCCGTTTACC
*IL-* *1β*	GGCTGCTCTGGGATTCTCTT	ATTTCACTGGCGAGCTCAGG
*IL-6*	GGCTATTCAGACAGCAGGGAGT	TGGTGTAAAGAGGACTGGGAAA
*IL-8*	GAATGGGTTTGCTAGAATGTG	ACTGTGAGGTAAGATGGTGGC

## Data Availability

The original contributions presented in this study are included in the article. Further inquiries can be directed to the corresponding author.
